# Supplementary material for: HIV-1 latency reversing agents converge on phosphoregulation of nuclear protein complexes
Source: bioRxiv. 2025 Nov 14:2025.11.13.688376. Preprint. [Version 1] doi: 10.1101/2025.11.13.688376 (PMC12642471; doi:10.1101/2025.11.13.688376)
Supplement: 1 [file NIHPP2025.11.13.688376V1-supplement-1.pdf]

bioRxiv preprint doi: https://doi.org/10.1101/2025.11.13.688376; this version posted November 14, 2025. The copyright holder for this preprint (which was not certified by peer review) is the author/funder, who has granted bioRxiv a license to display the preprint in perpetuity. It is made available under aCC-BY 4.0 International license.

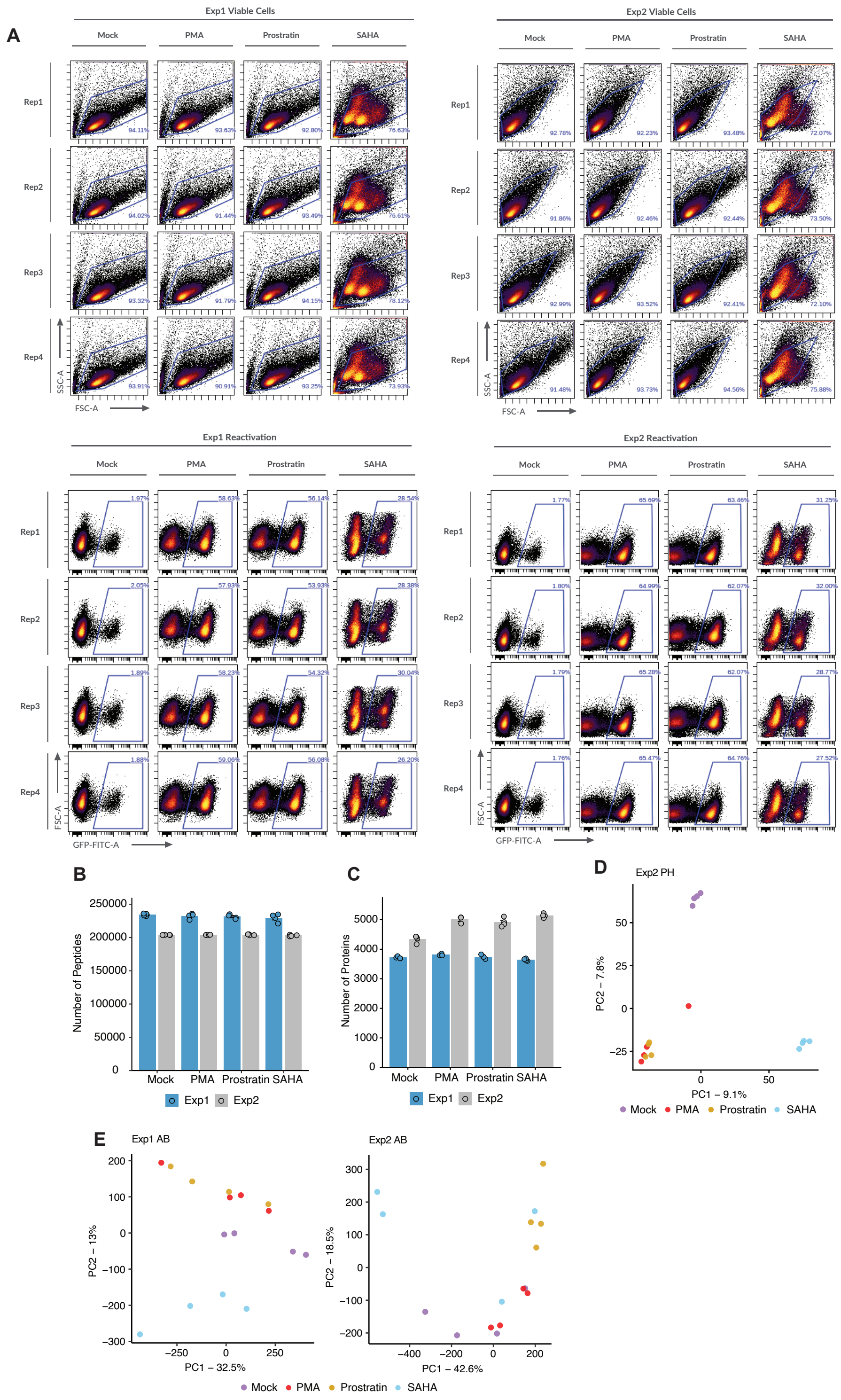

Figure S1. JLat flow cytometry and quality control results.
(A) Flow cytometry FSC-A vs. SSC-A and FITC-A (GFP) vs. FSC-A graphs, with conditions as the columns and replicates as the rows. The gating shows viable cells and the GFP (reactivation) percentage.

bioRxiv preprint doi: https://doi.org/10.1101/2025.11.13.688376; this version posted November 14, 2025. The copyright holder for this preprint (which was not certified by peer review) is the author/funder, who has granted bioRxiv a license to display the preprint in perpetuity. It is made available under aCC-BY 4.0 International license.

(B) Quantity of peptides detected in abundance proteomic data per LRA treatment and replicate across experiments 1 and 2.
(C) Number of high confidence (Localization Probability > 0.9) phosphorylated proteins identified from the phosphoproteomics analysis per LRA treatment and replicate across Exp1 & 2.
(D)PCA of experiment 2 phosphoproteomics(PH) data with all 16 samples, before removal of one of the PMA replicates.
(E) PCA of abundance(AB) proteomics data from Exp1 & 2.

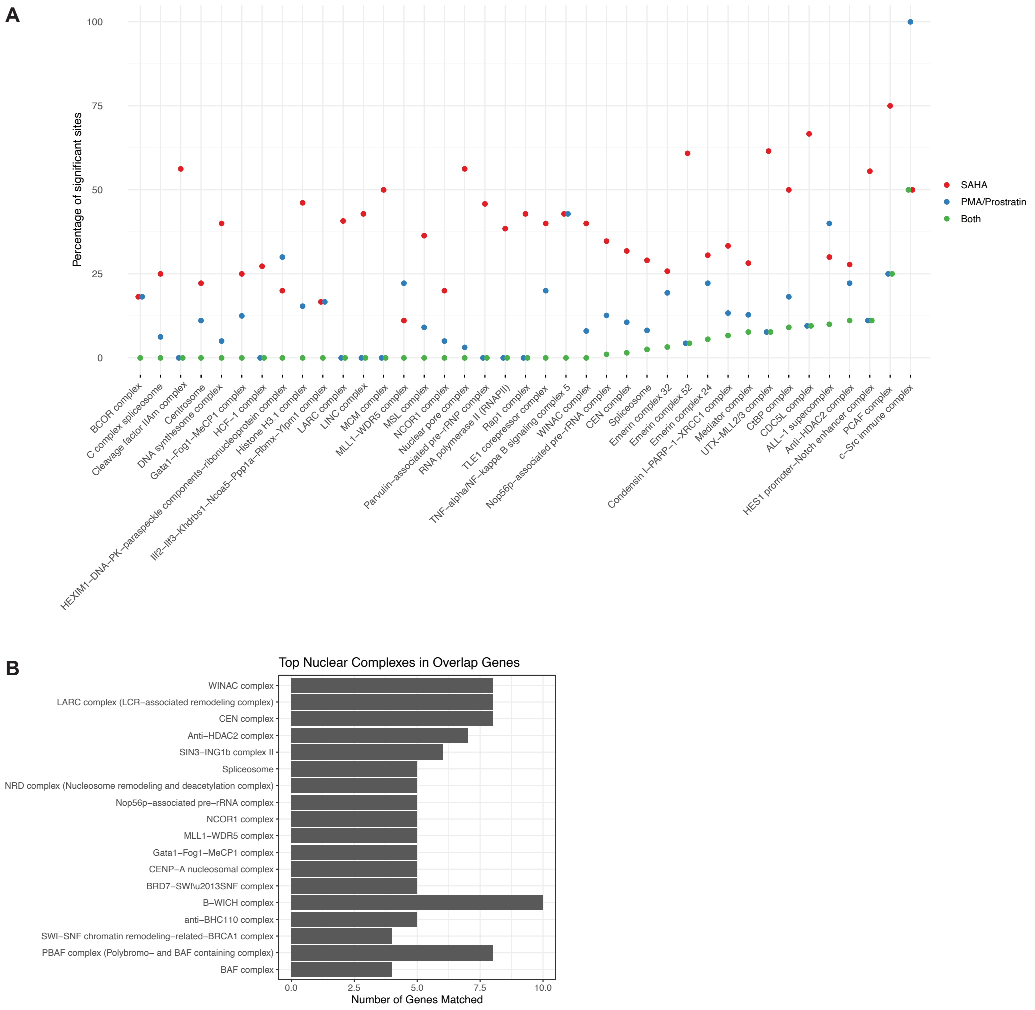

Figure S2. Overlap of phosphorylation sites between LRAs and with previously-reported knock-out screen dataset.
(A) Comparison of phosphosite regulation across the 39 nuclear protein complexes affected by SAHA and PKC agonists (PMA and Prostratin). Complexes with overlapping phosphosites include c-Src signaling, TFTC, HES1 promoter–Notch enhancer, anti-HDAC2, ALL-1 supercomplex, and CDC5L.
(B) Nuclear complexes involved in the overlap between LRA-regulated genes and a published HIV CRISPR screen by Hsieh et al..
